# Addressing rising knee injury and surgery rates with real-word data; the need for a clinical knee injury registry

**DOI:** 10.1016/j.jsampl.2024.100077

**Published:** 2024-10-04

**Authors:** Marc-Olivier Dubé, Kay M. Crossley, Andrea M. Bruder, Brooke E. Patterson, Melissa J. Haberfield, Adam G. Culvenor

**Affiliations:** a La Trobe Sport and Exercise Medicine Research Centre, School of Allied Health, Human Services and Sport, La Trobe University, Melbourne, Victoria, Australia; b Australian IOC Research Centre, La Trobe University, Melbourne, Victoria, Australia

## 1 Rising rates and underwhelming long-term outcomes of knee injuries: a recipe for trouble

The high and rapidly rising rate of traumatic knee injuries [1] has been likened to a global ‘sporting epidemic’ [2]. Incidence rates of anterior cruciate ligament (ACL) injury and meniscal tears worldwide (e.g., 80 [3] and 79 [4] per 100,000 Scandinavians, respectively) make them two of the most common knee injuries. Estimated ACL injury rates are even higher (85 per 100,000 people) for those at greater risk (i.e., 16–39 year olds) [5]. Australia stands atop the ACL incidence dais with as many as 91 per 100,000 people in those aged under 25 years [6]. Given that these figures are based on rates of ACL reconstruction (ACLR) surgery, the actual incidence of ACL injury is even greater.

Surgery remains the most common treatment for young people with an ACL rupture or isolated meniscal tear. However, randomised clinical trials failed to observe consistent superiority for early surgery compared to evidence-based rehabilitation programs and optional later/delayed surgery for ACL injuries [7] and isolated meniscal tears [8]. These findings are essential given that surgery drives most of the financial burden of traumatic knee injuries. In 2014–15 alone, AUD$142 million was spent on primary ACLR surgeries in Australia, a figure that will have grown in the last decade [6]. Attempts to change the paradigm to a first-line non-surgical approach have led to the emergence of new management strategies to facilitate healing of the native ACL (e.g., Cross Bracing Protocol) [9].

Regardless of the management approach, long-term outcomes of traumatic knee injuries are underwhelming. Forty-five percent of athletes do not return to competitive sport following ACL injury [10], one-third report unacceptable symptoms 1–2 years after ACLR [11] and, perhaps most devastating, one in five will reinjure their ACLR knee or suffer a new contralateral injury [12]. Disappointing figures also exist for meniscal tears – 17 ​% of athletes do not return to play after isolated meniscal repair [13] and 22 ​% do not return to pre-injury level after partial meniscectomy [14]. Those unsatisfying long-term outcomes contribute to poor quality of life for people with a history of ACL or meniscal injury compared to their uninjured peers [15]. Individuals who sustained an ACL injury or a combined ACL and meniscal injury have 4 to 6 times greater odds of developing post-traumatic OA, respectively [16]. Post-traumatic OA is a distinct form of OA associated with more disability years than insidious-onset OA in older adults given its development at a younger age [17]. Alarmingly, evidence suggests that OA is not simply a long-term outcome – up to one-third of ACL-injured individuals have signs of post-traumatic OA within the first 1–2 years postoperatively [18].

## 2 The current sex/gender disparity adds insult to injury

Gender (woman/girls/man/boy/transgender/non-binary related to socially constructed roles and behaviours) and sex (female, male, intersex determined by biological characteristics) factors may explain why a sex/gender ACL injury burden exists. Females experience a 2–6 times higher risk of ACL injury compared to males, when participating in the same sports [19]. With the recent explosion in women's and girl's participation in football codes, including soccer and Australian football, Australia has the world's highest rate of serious knee injuries (e.g. ACL rupture) in females. The annual number of ACL injuries is predicted to more than double by 2030 [1]. Females are not only at greater risk of injury, but have poorer outcomes post-injury and surgery compared to males. From a meta-analysis of 61 studies (16,555 participants), females face 25 ​% reduced odds of returning to sport than males, and 15–30 ​% worse knee-related long-term outcomes than their male counterparts [19]. After ACLR, females also have a 15 ​% higher risk of requiring revision surgery [20].

Historically, the higher injury risk and poorer outcomes for females were attributed to biological differences – *it's a women's problem; they simply have wider hips, and laxer ligaments and weaker muscles especially over different phases of the menstrual cycle* [21]. Although physical, anatomical, and hormonal factors may indeed play a role, we now understand that the reality is far more complicated!

Disparities in the gendered environment highlight that differences in outcomes for women may not simply be sex-related [22,23]. Gendered environment factors can influence women's outcomes across all socio-ecological levels (individual, interpersonal, organisation, policy) and at all points along the ACL injury spectrum—from childhood and pre-training to competition and rehabilitation [22,23]. These factors include access to, and experiences with, sport and training during childhood and the pre-competition period [22]. Indeed, women may not traditionally be exposed to the necessary level of conditioning to prevent such injuries [24] since strengthening, a core component of injury prevention programs, is often not perceived socially or culturally to be a high priority for women and girls [22]. Access to appropriate training spaces and equipment in a men-dominant environment may discourage women from accessing appropriate prevention and rehabilitation interventions [23]. Beyond injury prevention/rehabilitation factors, gendered social roles may impose additional barriers for women to invest sufficient time in their rehabilitation (typically primary carer role, home duties, pregnancy) to allow for a safe and successful return to pre-injury level of competition [19].

Contributing to this gender equity issue is the alarming underrepresentation of women in sports and exercise medicine research. For example, women/females only make up an estimated 10 ​% of participants in sport and exercise medicine clinical trials [25]. We do not know if research findings and associated recommendations could, or should, be applied to women – recent systematic reviews and consensus statements could not provide any gender/sex-specific evidence-informed treatments [15,26].

## 3 Call to action: a clinical knee injury registry to answer important questions

The unsatisfactory and unacceptable long-term outcomes and sex/gender disparity provide a clear and compelling case – we must do better. Future randomised controlled trials will contribute to the evidence base, but financial and practical constraints make clinical trials challenging to conduct, and their controlled nature often limits the implementation of findings to a broader clinical population. A registry can fill in some of those gaps. Understanding injury characteristics and treatment in large datasets across an entire community in real-world circumstances may lead to meaningful action. For example, detection of demographic factors (e.g., age, sex/gender, sport, region) associated with worse outcomes would ensure potential interventions/strategies are targeted and/or optimised. Perhaps most importantly, these interventions/strategies could then be evaluated by relevant stakeholders (e.g., government, healthcare system, sporting industry, professional organisations) with greater practicality and efficiency.

We need to follow the example of world leaders in the space. Scandinavian countries are pioneers in the field of surgical knee joint registries. The Norwegian knee ligament registry, the first of its kind, established in 2004, used the information collected on primary and revision ACL and PCL reconstructions to identify predictors of clinical outcomes and graft failure following ACLR [27]. In the Asia/Pacific region, New Zealand established the first and only real nationwide injury incidence data – in the form of an insurance registry [28]. Data from the New Zealand ACL registry, which includes more than 20,000 patients, has shown that patellar tendon grafts were associated with reduced risk of graft failure in females aged 15–20 years [29]. In Australia, the insights provided by the Australian Orthopaedic Association National Joint Replacement Registry (AOANJRR) have contributed to a reduction in the revision of joint replacement over time. It has been instrumental in improving outcomes of joint replacement surgery in Australia and has had an increasing global influence [30].

For many decades, an Australian ACLR registry – using hospital-linked data and input by surgeons – similar to those in Scandinavia and the AOANJRR, has been proposed [31]. However, such an ACLR registry would primarily focus on surgery-related outcomes. There is an opportunity to take a bigger step forward to understand treatment pathways and outcomes more broadly by including non-surgically managed patients and injuries other than ACL tears. For example, meniscal tears are a common knee injury at risk of longer-term OA that may not require surgery [8]. Registry data would help understand the relationship between individual characteristics, healthcare pathways, and long-term outcomes. For example, we do not know how patients navigate through healthcare following their knee injury, what informs the decision to undergo surgical or non-surgical management, and how this decision affects long-term outcomes.

However, the most significant hurdle in establishing such an initiative is large-scale data collection while avoiding duplication. Given that most patients who sustain a serious knee injury are likely to undergo diagnostic imaging, this pre-existing pathway could be used to streamline data collection and include all patients regardless of their management. Another alternative could be a combined approach: a surgical registry relying on data entered by surgeons and leveraging the established processes of the AOANJRR, alongside a non-surgical registry, potentially relying on data entered by various healthcare providers (surgeons, physiotherapists, sport and exercise physicians, etc.). Increased Medicare rebates for data entry into a non-surgical registry may help to incentivise data collection.

Establishing registries can be highly beneficial to patients and clinicians. Considering the rapidly increasing serious knee injury rates, there is a strong case for the creation of a knee injury registry collecting data in the Australian setting and going beyond what is captured in current ACLR registries. It's time to use real-world registry data to answer key questions about the incidence, management, and clinical course of traumatic knee injuries to help reshape knee health across Australia and the world.

## Author contributions

MOD and AGC wrote the manuscript, all co-authors revised the manuscript critically and approved the final version.

## Funding

MOD is a recipient of a *Fonds de Recherche du Québec – Santé (FRQS)* Postdoctoral Fellowship (327156). BEP is a recipient of an Australian Research Council (ARC) Early Career Industry Fellowship (IE230100135). AGC is a recipient of a National Health and Medical Research Council (NHMRC) of Australia Investigator Grant (GNT2008523). For the purposes of open access, the author has applied a CC-BY public copyright licence to any author accepted manuscript version arising from this submission.

## Declaration of competing interest

The authors declare the following financial interests/personal relationships which may be considered as potential competing interests: The authors are developing a new Australian Knee Injury Study, but otherwise have no conflicts of interest to declare.

## References

[1] Maniar N., Verhagen E., Bryant A.L., Opar D.A. (2022). Trends in Australian knee injury rates: an epidemiological analysis of 228,344 knee injuries over 20 years. Lancet Reg Health West Pac.

[2] Silvers H.J. (2009). Play at your own risk: sport, the injury epidemic, and ACL injury prevention in female athletes. J Intercoll Sport.

[3] Frobell R.B., Lohmander L.S., Roos H.P. (2007). Acute rotational trauma to the knee: poor agreement between clinical assessment and magnetic resonance imaging findings. Scand J Med Sci Sports.

[4] Peat G., Bergknut C., Frobell R., Jöud A., Englund M. (2014). Population-wide incidence estimates for soft tissue knee injuries presenting to healthcare in southern Sweden: data from the Skåne Healthcare Register. Arthritis Res Ther.

[5] Granan L.-P., Bahr R., Steindal K., Furnes O., Engebretsen L. (2008). Development of a national cruciate ligament surgery registry: the Norwegian National Knee Ligament Registry. Am J Sports Med.

[6] Zbrojkiewicz D., Vertullo C., Grayson J.E. (2018). Increasing rates of anterior cruciate ligament reconstruction in young Australians, 2000-2015. Med J Aust.

[7] Frobell R.B., Roos E.M., Roos H.P., Ranstam J., Lohmander L.S. (2010). A randomized trial of treatment for acute anterior cruciate ligament tears. N Engl J Med.

[8] Skou S.T., Hölmich P., Lind M., Jensen HP, Jensen C, Garval M (2022). Early surgery or exercise and education for meniscal tears in young adults. NEJM Evidence.

[9] Filbay S.R., Dowsett M., Jomaa M.C., Jane Rooney J., Sabharwal R., Lucas P. (2023). Healing of acute anterior cruciate ligament rupture on MRI and outcomes following non-surgical management with the Cross Bracing Protocol. Br J Sports Med.

[10] Ardern C.L., Taylor N.F., Feller J.A., Webster K.E. (2014). Fifty-five per cent return to competitive sport following anterior cruciate ligament reconstruction surgery: an updated systematic review and meta-analysis including aspects of physical functioning and contextual factors. Br J Sports Med.

[11] Patterson B.E., Culvenor A.G., Barton C.J., Guermazi A., Stefanik J.J., Crossley K.M. (2020). Patient-reported outcomes one to five years after anterior cruciate ligament reconstruction: the effect of combined injury and associations with osteoarthritis features defined on magnetic resonance imaging. Arthritis Care Res.

[12] Barber-Westin S., Noyes F.R. (2020). One in 5 athletes sustain reinjury upon return to high-risk sports after ACL reconstruction: a systematic review in 1239 athletes younger than 20 years. Sport Health.

[13] Blanchard E.R., Hadley C.J., Wicks E.D., Emper W., Cohen S.B. (2020). Return to play after isolated meniscal repairs in athletes: a systematic review. Orthop J Sports Med.

[14] D’Ambrosi R., Meena A., Raj A., Ursino N., Mangiavini L., Herbort M. (2023). In elite athletes with meniscal injuries, always repair the lateral, think about the medial! A systematic review. Knee Surg Sports Traumatol Arthrosc.

[15] Whittaker J.L., Culvenor A.G., Juhl C.B., Berg B., Bricca A., Filbay S.R. (2022). OPTIKNEE 2022: consensus recommendations to optimise knee health after traumatic knee injury to prevent osteoarthritis. Br J Sports Med.

[16] Poulsen E., Goncalves G.H., Bricca A., Roos E.M., Thorlund J.B., Juhl C.B. (2019). Knee osteoarthritis risk is increased 4-6 fold after knee injury - a systematic review and meta-analysis. Br J Sports Med.

[17] Lie M.M., Risberg M.A., Storheim K., Engebretsen L., Øiestad B.E. (2019). What’s the rate of knee osteoarthritis 10 years after anterior cruciate ligament injury? An updated systematic review. Br J Sports Med.

[18] Culvenor A.G., Collins N.J., Guermazi A., Cook J.L., Vicenzino B., Khan K.M. (2015). Early knee osteoarthritis is evident one year following anterior cruciate ligament reconstruction: a magnetic resonance imaging evaluation. Arthritis Rheumatol.

[19] Bruder A.M., Culvenor A.G., King M.G., Haberfield M., Roughead EA, Mastwyk J (2023). Let’s talk about sex (and gender) after ACL injury: a systematic review and meta-analysis of self-reported activity and knee-related outcomes. Br J Sports Med.

[20] Tan S.H.S., Lau B.P.H., Khin L.W., Lingaraj K. (2016). The importance of patient sex in the outcomes of anterior cruciate ligament reconstructions: a systematic review and meta-analysis. Am J Sports Med.

[21] Hewett T.E., Myer G.D., Ford K.R. (2006). Anterior cruciate ligament injuries in female athletes: Part 1, mechanisms and risk factors. Am J Sports Med.

[22] Parsons J.L., Coen S.E., Bekker S. (2021). Anterior cruciate ligament injury: towards a gendered environmental approach. Br J Sports Med.

[23] Bruder A.M., Crossley K.M., Donaldson A., Mosler A.B. (2021). Through the athlete lens: a novel study exploring the perspectives and experiences of injury prevention practices in women playing elite Australian Football. Braz J Phys Ther.

[24] Caspersen C.J., Pereira M.A., Curran K.M. (2000). Changes in physical activity patterns in the United States, by sex and cross-sectional age. Med Sci Sports Exerc.

[25] Cowan S.M., Kemp J.L., Ardern C.L., Thornton J.S., Rio E.K., Bruder A.M. (2023). Sport and exercise medicine/physiotherapy publishing has a gender/sex equity problem: we need action now. Br J Sports Med.

[26] Culvenor A.G., Girdwood M.A., Juhl C.B., Patterson B.E., Haberfield M.J., Holm P.M. (2022). Rehabilitation after anterior cruciate ligament and meniscal injuries: a best-evidence synthesis of systematic reviews for the OPTIKNEE consensus. Br J Sports Med.

[27] Martin R.K., Wastvedt S., Pareek A., Persson A., Visnes H., Fenstad A.M. (2022). Predicting anterior cruciate ligament reconstruction revision: a machine learning analysis utilizing the Norwegian knee ligament register. J Bone Joint Surg Am.

[28] Gianotti S.M., Marshall S.W., Hume P.A., Bunt L. (2009). Incidence of anterior cruciate ligament injury and other knee ligament injuries: a national population-based study. J Sci Med Sport.

[29] Tiplady A., Love H., Young S.W., Frampton C.M. (2023). Comparative study of ACL reconstruction with hamstring versus patellar tendon graft in young women: a cohort study from the New Zealand ACL registry. Am J Sports Med.

[30] Steiger RN de, Graves S.E. (2019). Orthopaedic registries: the Australian experience. EFORT Open Reviews.

[31] Orchard J.W. (2011). Why does Australia have a higher rate of knee reconstruction surgery than New Zealand (and Scandinavia) and what can we do about it?. J Sci Med Sport.

